# Next-Generation Sequencing–Guided Treatment of BRCA2-Mutant Metastatic Uterine Leiomyosarcoma With Poly(ADP-ribose) Polymerase Inhibitor Therapy

**DOI:** 10.1200/PO-24-00401

**Published:** 2024-12-12

**Authors:** Cameron Kirkendoll, Richard Mansour, Shiva Jashwanth Gaddam

**Affiliations:** ^1^ Department of Hematology and Oncology, LSU Health Sciences, Shreveport, LA

## Introduction

Sarcomas represent a class of malignancies that are derived from mesenchymal tissue. Soft tissue sarcomas (STSs) are a subset of mesenchymal neoplasms that comprise roughly 1% of all adult malignancies.^1^ Leiomyosarcomas (LMSs) are an aggressive subtype of STS, most often found in the abdomen and pelvis. Uterine leiomyosarcomas (uLMSs) are still rarer, with an annual incidence of only 0.35-0.65 per 100,000 women in the United States.^2^ Since uLMSs are considered aggressive, early intervention is imperative. Surgery is considered first line for all localized, clinically resectable sarcomas. Chemotherapy may be used as neoadjuvant or adjuvant therapy. However, treating advanced uLMSs has proven difficult. The SEER database has found that once the disease progresses to distant metastasis, the 5-year relative survival rate is only 12%.^3^ Once uLMSs have metastasized, the prospect of attaining long-term survival is very low.^4^ After progression on first-line therapy, treatment options are limited and outcomes with second-line therapy and beyond are dismal.

Precision oncology and targeted therapies have made remarkable impact on outcomes in a wide range of malignancies. Further exploration into their effectiveness against uLMSs is needed. With the use of next generation sequencing (NGS), potential targets of therapy can be identified. The *BRCA1/2* mutations are well-known drivers of various cancers. Poly(ADP-ribose) polymerase inhibitors (PARPi) are a drug class that targets PARP 1, effectively disrupting the tumor's self-repair mechanism and leading to apoptosis.^5^ This drug class has shown synthetic lethality in tumor cells that lack *BRCA1/2* activity. One case series found that approximately 10% of uLMS tumors carried a *BRCA2* mutation, making them potential candidates for PARPi therapy.^1^

In this report, we present a case of a patient with metastatic uLMS, positive for a somatic *BRCA2* mutation who showed significant reduction in tumor size after initiation of PARPi therapy.

## Case Presentation

**FIG 1.**
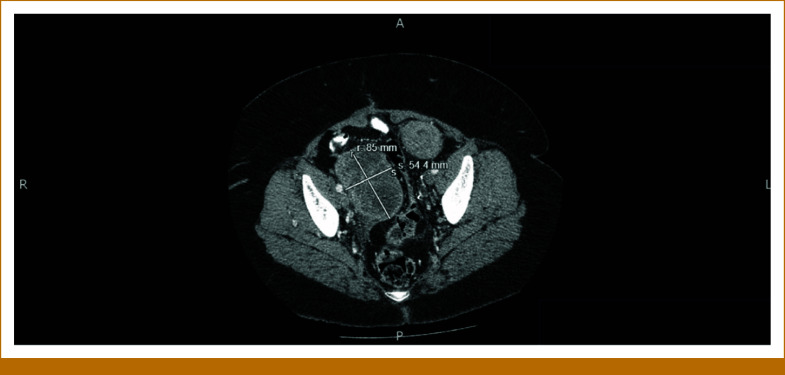
Approximately week 83; before initiating second round of gemcitabine/docetaxel.

**FIG 2.**
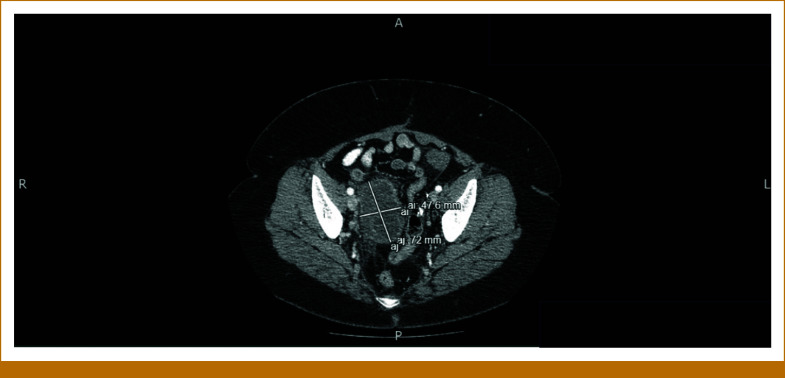
Approximately week 90; 7 weeks into second round of gemcitabine/docetaxel treatment.

**FIG 3.**
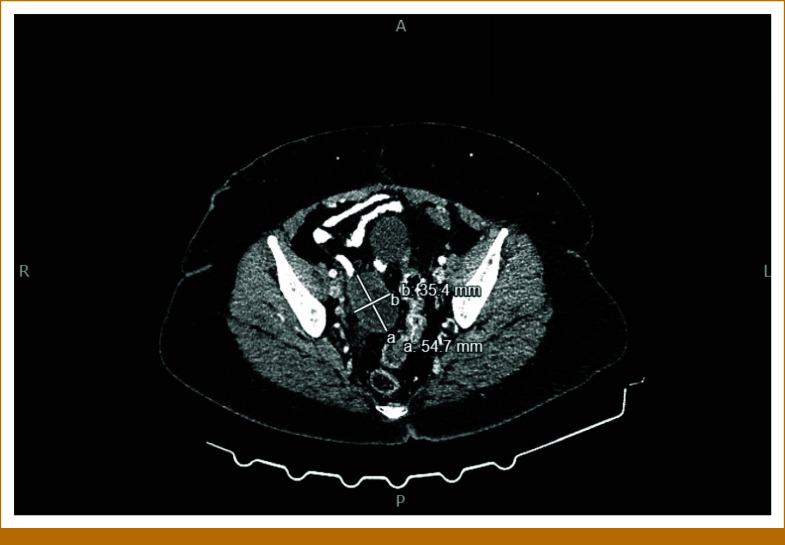
Approximately week 109; roughly 1 week before starting niraparib (poly(ADP-ribose) polymerase inhibitor).

**FIG 4.**
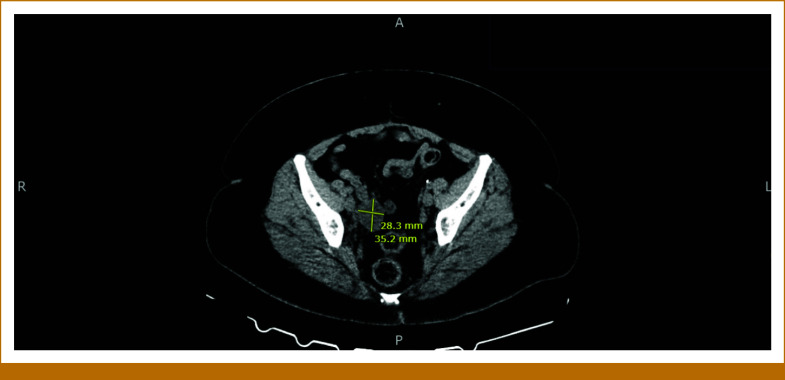
Approximately week 125.

**FIG 5.**
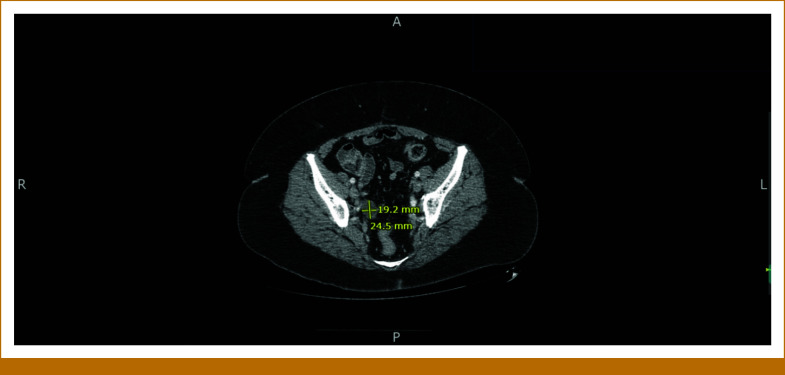
Approximately week 157.

**FIG 6.**
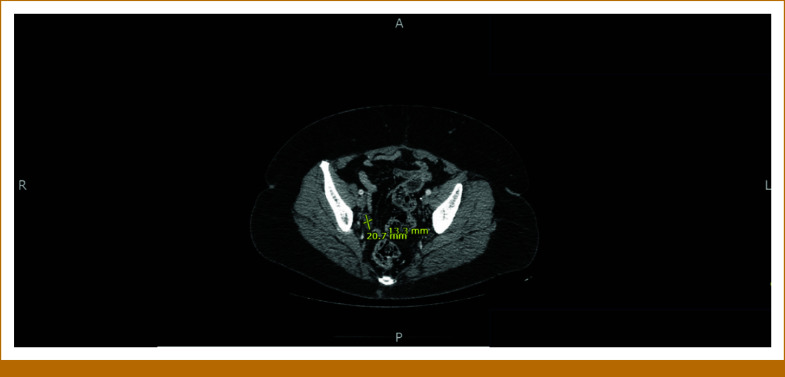
Approximately week 214.

The patient is a 58-year-old woman who presented to her gynecologist with 10 days of postmenopausal bleeding. Pelvic ultrasound revealed an enlarged uterus with multiple focal masses, consistent with fibroids, and a thickened endometrial stripe. After undergoing radical hysterectomy, she was referred to our oncology clinic. The pathology report was positive for uterine leiomyosarcoma (uLMS). The tumor invaded the myometrium, sparing the ovaries. A left-sided pelvic nodule biopsy was positive for metastatic disease, along with small bowel mesentery and a right-lung mass. The tumor size was 31.5 cm × 23.2 cm × 14 cm, weighing roughly 5,000 g. The final pathologic stage was pT3a, pNx, M1 (lung biopsy). She was informed that this cancer carries a poor prognosis, and the goal of therapy is palliative with aim to control the disease for as long as possible. She was started on gemcitabine and docetaxel for a total of six cycles (21-day cycles), scheduled during weeks 4-23. Restaging scans in week 16 showed improved lung nodules. Repeat computed tomography (CT) scans during week 60 showed stable disease. However, in week 80, a repeat CT of the chest, abdomen, and pelvis (CT CAP) showed interval development of right upper and bilateral lower lobe soft tissue nodules in the lungs, along with interval development of multiple large retroperitoneal and pelvic masses. She was restarted on the same regimen of gemcitabine and docetaxel, scheduled during weeks 83-101. See Figure 1 for imaging before the second round of gemcitabine and docetaxel was initiated. Repeat CT CAP in week 90, displayed in Figure 2, showed improvement in most lung lesions, as well as improvement in the left-sided pelvic mass. Tissue NGS (Tempus xT 648 gene panel) was obtained in week 81, revealing a *BRCA2* copy number loss (unknown whether biallelic or monoallelic), *TP54* frameshift mutation, *RB1* and *TOP2A* mutations, with a high tumor mutation burden (TMB) of 10 m/MB. She did not have any germline mutations. Although TMB of 10 m/MB would qualify her for pembrolizumab per the tumor agnostic approval, it was deemed not to be ideal in her case given that the Keynote-158 trial that got this approval did not include patients with LMS.^6^ The *BRCA2* mutation provided an option for a PARP inhibitor (PARPi). She was started on niraparib 300 mg once daily in week 110. See Figure 3 for imaging completed before initiating niraparib. Figures 4 and 5 demonstrate continued response on niraparib with no significant adverse events. Her most recent CT CAP in week 215, shown in Figure 6, showed stable disease.

The Institutional Review Board determined that the proposed activity is not research involving human subjects as defined by DHHS and US Food and Drug Administration regulations.

## Discussion

There is currently a lack of high-quality evidence to define the standard of care in the second-line setting and beyond. Whenever possible, enrollment in clinical trials is strongly recommended; however, a significant proportion of patients lack access to these opportunities. In such cases, treatment options include pazopanib, trabectedin, other chemotherapy agents, and biomarker-directed therapies. Nonetheless, clinical responses to these treatments remain modest, underscoring the unmet need for therapies that offer more durable outcomes.

Precision oncology using targeted therapies has substantially enhanced survival results in a multitude of cancer types. Most targeted treatments are usually either small-molecule inhibitors or monoclonal antibodies that act by binding either to a cell surface receptor or to an intracellular molecule. The small molecule inhibitors are particularly effective in decreasing tumor cell burden by downregulating the cellular pathways that either are promoting cell division or are preventing cell death. PARPis are small molecule inhibitors that have been shown to use the mechanism of synthetic lethality to induce apoptosis and thereby, tumor regression in select patients.

Our DNA continuously incurs damage throughout a cell's life, and faulty DNA damage repair is considered as a hallmark of cancer.^7^ A double-stranded break (DSB) is one of the critical DNA damage effects, and the cell's stability and sustainability is dependent on the repair of these DSBs by a series of intercalating events called homologous recombination repair (HRR).^8^ Gene mutations (change in the gene DNA sequence which may or may not lead to complete loss of function) or gene losses (complete loss of function, by various mechanisms such as gene deletion and silencing) in any of the HRR genes, such as *BRCA1/BRCA2*, *PALB2*, and *ATM*, lead to HRR deficiency (HRRd). DNA also incurs single-stranded breaks (SSBs), which are repaired by PARP. If PARP activity is decreased, the SSBs accumulate and ultimately lead to DSBs. Thus, in HRRd cells, inhibition of PARP accrues increasing number of DSBs, which eventually induces cell death. This has been described as the theory of synthetic lethality, which signifies that loss of either *BRCA* or PARP is not detrimental to the cell's life, but coexistent loss of both is lethal. PARPis were developed using this rationale.

PARPis have been proven to be effective in multiple cancers with HRRd (ie, breast, ovarian, pancreas, and prostate cancers). Their efficacy in other malignancies is upcoming. Our case demonstrates the effectiveness of PARPi therapy in *BRCA2*-mutant uLMS. uLMS is an aggressive mesenchymal malignancy notorious for recurrence and metastasis. Survival rates are poor, especially with unresectable disease/advanced stage. Limited treatment options are available given the lack of clinical trials because of rarity of the disease. Immunotherapy has been tried in an attempt to induce better responses, but without much success. Hence, it is vital to investigate targeted therapies in uLMS.

Several case reports have explored the use of olaparib in treating *BRCA2*-mutant LMS. One report describes its effectiveness in recurrent uLMS with a *BRCA2* mutation, while another highlights its role in managing *BRCA2*-mutant colorectal LMS.^9,10^ Additionally, olaparib has been used in a patient with metastatic uLMS, where deep deletions in *BRCA2*, *TP53*, and *PTEN* were identified.^11^ Our case demonstrates the effectiveness of niraparib therapy in *BRCA2*-mutant uLMS.

PARPi is a promising option in patients with uLMS with *BRCA* mutations. Although *BRCA* mutations were found in approximately only 10% of patients with uLMS, PARPi remains an important development in the treatment of this disease given the tumor's aggressive growth and high mortality.^1^ In this case, NGS was used to identify a *BRCA* mutation in the patient, which was then targeted for treatment, resulting in an impressive reduction in tumor size and improvement in the patient's quality of life. A phase II clinical trial investigated the use of olaparib and temozolomide in treating advanced uLMS, and the published results showed that the median progression-free survival was significantly improved in HRRd patients versus HRR-proficient patients: 11.2 versus 5.4 months, *P* = .05.^12^ Another phase II/III trial has started recruiting patients to examine the effects of olaparib and temozolomide against the current standard of treatment after progression on chemotherapy (ClinicalTrials.gov identifier: NCT05432791). This case is worth discussing because while we await further prospective trials, there remains a paucity of data available on the use of PARPi in uLMS. It has also shown the benefit of using NGS to recognize genetic aberrations that would qualify certain patients as candidates for targeted therapies.

In conclusion, uLMSs represent a rare subtype of mesenchymal malignancies that do not usually portend a good prognosis, especially in the context of distant metastasis, where treatment options are limited. Our case report has shown the impressive results of PARPi therapy used in a patient with a somatic *BRCA2* mutation. To our knowledge, this is the first case demonstrating the utility of niraparib in such patients. Further prospective clinical trials are needed to investigate the potentially robust and durable effects of PARPi therapy in *BRCA* mutation–positive uLMS.

## Data Availability

The health information in this report was collected throughout the patient’s course of care. The patient has consented to their health information being used in this report.
